# Modification of Cotton and Leather Surfaces Using Cold Atmospheric Pressure Plasma and TiO_2_-SiO_2_-Reduced Graphene Oxide Nanopowders

**DOI:** 10.3390/ma16041397

**Published:** 2023-02-07

**Authors:** Dragoș-Viorel Cosma, Cristian Tudoran, Maria Coroș, Crina Socaci, Alexandra Urda, Alexandru Turza, Marcela-Corina Roșu, Lucian Barbu-Tudoran, Ioana Stanculescu

**Affiliations:** 1 Department of Mass Spectrometry, Chromatography and Applied Physics, National Institute for Research and Development of Isotopic and Molecular Technologies (INCDTIM), 67-103 Donat Street, 400293 Cluj-Napoca, Romania; 2 Cetatea, National Institute for Research and Development of Isotopic and Molecular Technologies (INCDTIM), 67-103 Donat Street, 400293 Cluj-Napoca, Romania; 3 Electron Microscopy Center “Prof. C. Crăciun”, Faculty of Biology & Geology, “Babeș-Bolyai” University, 5-7 Clinicilor Street, 400006 Cluj-Napoca, Romania; 4 Electron Microscopy Integrated Laboratory, National Institute for Research and Development of Isotopic and Molecular Technologies, 67-103 Donat Street, 400293 Cluj-Napoca, Romania; 5 Analytical Chemistry and Physical Chemistry Department, Faculty of Chemistry, University of Bucharest, Regina Elisabeta, no. 4-12, 030018 Bucharest, Romania; 6 Horia Hulubei National Institute of Research and Development for Physics and Nuclear Engineering, 30 Reactorului Str., 077125 Magurele, Romania

**Keywords:** TiO_2_-SiO_2_, reduced graphene oxide, cold atmospheric pressure plasma, photocatalytic activity, self-cleaning

## Abstract

Surface modification of textile fabrics and leathers is very versatile and allows the products quality improvement. In this work, cotton and leather substrates were pre-treated with cold atmospheric pressure plasma (CAPP) and further coated with TiO_2_-SiO_2_-reduced graphene oxide composites in dispersion form. By using a Taguchi scheme, this research evaluated the effect of three significant parameters, i.e., the pre-treatment with CAPP, organic dispersion coating and TiO_2_-SiO_2_-reduced graphene oxide (TS/GR) composites, that may affect the morpho-structural properties and photocatalytic activity of modified cotton and leather surfaces. The characteristics of cotton/leather surfaces were evaluated by morphological, structural, optical and self-cleaning ability using scanning electron microscopy with energy dispersive X-ray spectroscopy (SEM/EDX), X-ray powder diffraction (XRD), attenuated total reflection–Fourier Transform Infrared spectroscopy (ATR-FTIR) and UV–Vis spectroscopy. The self-cleaning performance of the obtained cotton and leather samples was evaluated by photocatalytic discoloration of berry juice surface stains under UV light irradiation for 12 h. The successfulness of coating formulations was proven by the SEM analysis and UV–Vis spectroscopy. The XRD patterns and ATR-FTIR spectra revealed the cellulose and collagen structures as dominant components of cotton and leather substrates. The CAPP treatment did not damage the cotton and leather structures. The photocatalytic results highlighted the potential of TiO_2_-SiO_2_-reduced graphene oxide composites in organic dispersion media, as coating formulations, for further use in the fabrication of innovative self-cleaning photocatalytic cotton and leather products.

## 1. Introduction

Currently, new challenges to develop higher-value-added textiles and leather products constantly appear due to market dynamics and global competitiveness. To satisfy the requirements of customers, various creative innovation methods for obtaining effective multifunctional materials are approached. In this context, significant attention in the specific scientific literature is attributed to nanoscience/nanotechnology [1,2,3,4], resulting in textile and leather products with many advantageous features, such as electrical and thermal conductivity, flame retardancy, hydrophobicity/oleophobicity, ultraviolet-blocking, antistatic, self-cleaning, microbicide and the like. As recently stated by MM Rashid et al. [5] in their comprehensive overview on TiO_2_/textile composites, the most studied nanomaterials used for textile surface modifications are Ag, TiO_2_, SiO_2_, ZnO, carbon nanotubes, graphene and reduced graphene oxide. These nanomaterials could provide multifunctional properties to textile fibers, such as UV protection, photocatalytic self-cleaning, electrical conductivity, thermal stability, antimicrobial activity and more. Application of nanotechnology in the leather industry has been highlighted in his review by L Muthukrishnan [3], showing that a variety of nanomaterials, such as Ag, Au, Pd, Se, Cu, Pt, TiO_2_, SiO_2_, ZnO, CuO, Fe_3_O_4_, polymers, bimetals and graphene derivatives, have been applied at various stages of manufacturing to fabricate high-value leather products. More recently, graphene-like materials have been successfully used for textile fabrics proving electrical conductivity, flame retardant, antistatic, hydrophilic/hydrophobic, ultraviolet-blocking, self-cleaning, antimicrobial and biocompatible properties [6,7], as well as in the leather sector [8,9]. Other new approaches, such as irradiation processes, became more and more popular as chemical-free surface-modification techniques, improving the dye uptake, wettability, hydrophobicity, fastness properties, flammability, adhesion of coatings or stain, etc. By considering technical properties, i.e., ease of handling and high treatment speed, and economical–environmental properties, i.e., energy, water and chemical consumption factors, using irradiation methods opens up new opportunities as green alternatives to the wet-chemical processing of textiles and leathers [10,11]. According to previous research studies summarized by S. Shahidi and J. Wiener [10], corona discharges (atmospheric pressure plasma), ultraviolet treatments, microwaves, lasers and gamma irradiations have been already successfully applied in the textile and leather sector. Among them, plasma techniques are applied on areas of textiles and leather to improve their surface features, such as hydrophilicity/hydrophobicity, dyeability, antistatic, antimicrobial and disinfectant. The mainly used plasma processes for modifying the surface properties of textile and leather materials are low-pressure plasma and atmospheric pressure (cold) plasma. In fact, the high-efficiency plasma cleaning, activation and nanocoating of surfaces is attributed to the interaction between substrate and reactive species in plasma, such as primary plasma species (electrons, ions, radicals and dissociated molecules), reactive oxygen (ROS) and nitrogen species (RNS) and ultraviolet photons. However, the nature of the substrate and the process conditions (working gas, power, frequency and treatment time) are important factors that influence the plasma efficacy [12,13].

Inspired by these findings, this work presents an evaluation of the morphological, structural and optical properties of cotton samples in parallel with those of leather samples using TiO_2_-SiO_2_-reduced graphene oxide nanopowders in organic dispersion media, organosilicon compounds, plant-based polymers and biodegradable solvent as coating formulations and CAPP treatments, using Ar and Ar/O_3_ as gas plasma. Many papers [14,15,16,17,18,19,20,21,22,23,24] have already reported the modification of textiles, but only some of the literature’s data report about improved leather surfaces [25,26,27,28] using TiO_2_/SiO_2_, TiO_2_/graphene oxide and SiO_2_/graphene oxide. Moreover, we have found only one prior report on TiO_2_/SiO_2_/GO nanocomposites deposited onto the polyester/cotton fabric surface in order to impart a self-cleaning function [29] as well as on the combination of atmospheric pressure plasma treatment and nano-finishing treatment using a TiO_2_-SiO_2_ nanocomposite on the surface properties of leather [30]. Generally, reported research works individually investigated the influential factors. By using a Taguchi scheme, this research evaluated the effect of three significant parameters (i.e., the pre-treatment with CAPP, organic dispersion coating and TiO_2_-SiO_2_-reduced graphene oxide) that may affect the morpho-structural properties and photocatalytic activity of modified cotton and leather surfaces. The Taguchi experimental design allows comparing the effects of several variables, together with the interactions between them, with a reduced number of experiments [31]. Moreover, this study was performed on cotton and leather surfaces in parallel. The study provides important data regarding the improvement of textile and leather surfaces in order to develop value-added eco-friendly products.

## 2. Materials and Methods

### 2.1. Reagents and Materials

The chemicals used for graphene oxide synthesis were the following: natural graphite (Sigma Aldrich, St. Louis, Mo, USA), sulfuric acid (H_2_SO_4_, 95–97%, Merck, Darmstadt, Germany), potassium persulfate (K_2_S_2_O_8_, Merck, Darmstadt, Germany), phosphorus (V) oxide (P_2_O_5_, Merck, Darmstadt, Germany), sodium nitrate (NaNO_3_, Honeywell and Sigma-Aldrich, Gurgaon, India), potassium permanganate (KMnO_4_, Merck, Darmstadt, Germany), hydrogen peroxide (H_2_O_2_, Merck, Darmstadt, Germany), hydrochloric acid (HCl, 36~38%, Merck, Darmstadt, Germany) and double-distilled water. TiO_2_ P25 nanoparticles from Degussa and SiO_2_ Aerosil 200 from Evonik (Essen, Germany) were used for the preparation of mixed TiO_2_-SiO_2_ powder. Acetylacetone and ethanol as organic dispersing agents were purchased from Merck, Darmstadt, Germany and Fluka. Organosilicon compounds namely, octamethylcyclotetrasiloxane, D4 and decamethylcyclopentasiloxane and D5 and biodegradable ethyl-lactate (Et-L) solvent were purchased from Alfa Aesar, Kandel, Germany. Alginic acid sodium salt (NaAlg) and gum rosin (GRos) were obtained from Aldrich, Norwich, United Kingdom and Aveiro, Portugal. Calcium chloride (CaCl_2_, Alfa Aesar, Kandel, Germany) was used to promote physical crosslinking and form water-insoluble calcium alginate (CaAlg) on cotton and leather surface.

Bleached 100% cotton woven fabric with the weight of 168 g/m^2^ was used for all experiments. The sheepskin leather surfaces were finished according to the classical technologies by spraying a base coat of acrylic resins with water-based casein pigment and a topcoat of water-based nitrocellulose emulsion [25]. The cotton and leather materials were provided by the National Research Institute for Leather and Textiles, Bucharest, Romania.

### 2.2. Synthesis of Graphene Oxide (GO)

Graphene oxide (GO) was synthesized from natural graphite powder by our improved version of Hummer’s method [32]. In the first (pre-oxidation) stage, 4.6 g of graphite was slowly added into a tri-component mixture of concentrated H_2_SO_4_ (8 mL), K_2_S_2_O_8_ (2.5 g) and P_2_O_5_ (2.5 g), under constant magnetic stirring at 80 °C. In the second stage, after water cleaning and vacuum filtration, the resulting pre-oxidized graphite (3.5 g) was mixed with 1.6 g of NaNO_3_ and 60 mL of H_2_SO_4_ under continuous stirring in an ice bath. Subsequently, 8 g of KMnO_4_ was gradually added in the oxidation process, keeping the temperature less than 20 °C to prevent overheating and explosion. Following a dilution step with double-distilled water, the resulting mixture was treated with 3% H_2_O_2_ (till effervescence was reduced) to transform the residual oxidants. For further purification, the brown suspension was rinsed with 1% HCl solution and double-distilled water several times. The reaction product was purified through dialysis and then was ultrasonicated for 90 min to achieve the complete exfoliation. Finally, the GO powder was obtained by freeze drying.

### 2.3. Preparation of Mixed TiO_2_-SiO_2_ and TiO_2_-SiO_2_/Reduced Graphene Oxide Nanopowders

The mixed TiO_2_-SiO_2_ nanopowder was prepared through a combined physical–mechanical/thermal method. TiO_2_ (10 g) and SiO_2_ (0.6 g) nanopowders were dispersed into a solution of 0.5 g of acetyl–acetone in 20 mL of double-distilled water under magnetic stirring at 300 rpm. After homogenization, the mixture of oxides was dried on a plate at 50 °C and then annealed at 450 °C for 1 h, in air. TiO_2_-SiO_2_/reduced graphene oxide composite (10:1 weigth ratio) was prepared by ultrasonic dispersion of mixed TiO_2_-SiO_2_ nanopowder and graphene oxide into 20% ethanol solution, drying and annealing at 300 °C in argon atmosphere. The obtained TiO_2_-SiO_2_ powders were denoted TS and TS/GR, where GR represents the reduced graphene oxide, with partial reduction of the functional groups on the GO surface, obtained as a result of the thermal treatment.

### 2.4. Preparation of Treated Cotton and Leather Samples

The standardized Taguchi-based experimental design, an L_6_, 3^2^ × 2^1^ scheme, with three columns and six rows, ref. [31], was used in order to find the optimal condition for obtaining improved performance characteristics of final cotton and leather samples (Table 1). The levels for controlling factors were for factor A: no plasma pre-treatment of sample surfaces, plasma pre-treatment in Ar and Ar/O_3_ atmosphere; for factor B: Et-L, D4/D5/Et-L mixture (D4:D5:Et-L = 2:2:1 vol. ratio) and GRos/Et-L and NaAlg/H_2_O mixture (0.5% sol. GRos in Et-L:0.5% aqueous sol. NaAlg = 2:1 vol. ratio); and for factor C: TS and TS/GR composites.

The described approach was applied for both cotton and leather samples of 15 × 10 cm size. An in-house laboratory-made plasma generator unit from INCDTIM was used for treatment of sample surfaces prior to deposition of coating formulations. The treatment system contains a newly designed plasma applicator head, based on the principle of the swept cylindrical dielectric barrier discharge plasma, fed by a high voltage power source (protected by patent RO133593A0) [33].

The plasma working parameters were as follows: nominal input power: 50 W; supply frequency: up to 10 kHz; plasma type: discharge with dielectric barrier in pulses; plasma kinetic temperature: <50 °C; plasma current density: 12 mA cm^−2^.

Based on the information from the review of R.A. Jelil [12] and also on our research group’s experience, the plasma pre-treatment time was established to 3 min for cotton and 9 min for leather surfaces, because the reactive gas particles easily and deeply penetrate the looser, airier textile than the dense fibrous leather structures. Argon was chosen as working gas because it is the most common gas to generate plasma due to its advantages: chemical inertness, large ionization energy and relative low cost as compared to other noble gases [12]. Oxygen-containing plasma, by using a mixture of Ar and O_3_ (Ar/O_3_ flow ratio of 10/1), is expected to produce more severe ablation or etching of the cotton and leather surfaces due to the powerful oxidizing effect of ozone.

The deposition of the composite suspensions on cotton and leather surfaces was performed via classical spraying using a commercially available air brush kit, nozzle diameter of 0.2 mm and air pressure of 2 bars. The distance between substrates and jet nozzle was kept constant at 15 cm. All final cotton (denoted Exp 1-CT…Exp 6-CT) and leather (denoted Exp 1-LH…Exp 6-LH) samples were dried at room temperature. The raw cotton and leather materials were denoted as control cotton (CT) and control leather (LH).

### 2.5. Characterization of Cotton and Leather Samples

Hitachi SU-8230 scanning electron microscope (Tokyo, Japan) operated at 30 kV was used to observe the morphology and elemental composition of final cotton and leather samples. The crystalline phases were identified by X-ray powder diffraction (XRD) on Bruker D8 Advance diffractometer (Karlsruhe, Germany) using CuK_α1_ radiation (λ = 1.540598 Å). The diffraction peaks were identified from ICDD’s Powder Diffraction File (PDF) database with Match! version 1.11k software. Attenuated total reflection–Fourier transform infrared spectroscopy (ATR-FTIR) was recorded on FT-IR Jasco 6100 spectrometer (Jascp International Co, Ltd., Tokyo, Japan) to determine the chemical structure of the cotton and leather samples. Investigation of the optical absorption properties of all samples was carried out using a Jasco V-570 Spectrophotometer (Jasco International Co., Ltd., Tokyo, Japan).

### 2.6. Evaluation of Self-Cleaning Photocatalytic Activity

Self-cleaning characterization of cotton and leather samples was performed by photo discoloration of berry juice stains from their surface. The samples of dimensions 2 × 5 cm^2^ were 3 times sprayed (using the aerograph) with 5 mL of natural berry juice obtained by squeezing fruits (without dilution). After drying in air, the stained samples were exposed to UV light using the Photoreactor Luzchem LZC-4V (Ottawa, Ontario, Canada) equipped with 14 Blacklight Blue (8 W) LEDs that emit UV-A light at 352 nm. The degree of discoloration of berry juice stains was measured (at a certain wavelength: λ = 535 nm) by Jasco V-570 spectrophotometer, and the efficiency of degradation (%) was calculated following the relation Equation (1):

(1) $$\mathrm{EF}\;\left(\%\right)\;=\;\left(A_{0}-A_{t}\right)\;\times 100/A_{0},$$

where A_t_ and A_0_ represent the maximum absorbance intensity at time t and at initial time (t = 0).

## 3. Results and Discussion

### 3.1. Scanning Electron Microscopy Analysis

**Figure 1:**
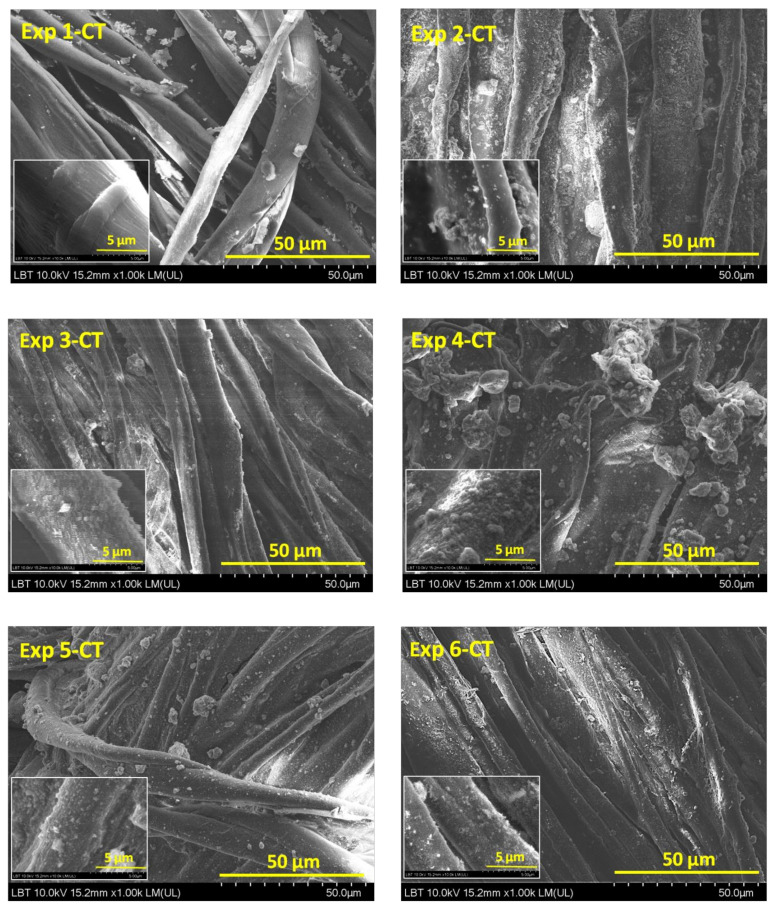
SEM images of Exp 1-CT…Exp 6-CT samples with magnification power of ×1000.

SEM analysis can provide information on the morphology of cotton and leather surfaces and the differences among samples before and after treatment. SEM images (Figure 1) showed the typical longitudinal stripes of cotton fibers (with a diameter in the range of 3–15 μm). A lower quantity of particles was deposited on the Exp 1-CT surface compared with those of Exp 2-CT…Exp 6-CT. Comparing Exp 1-CT with Exp 2-CT, a better anchoring of particles was observed for Exp 2-CT. The only difference between the two samples is the presence of reduced graphene oxide in the coating formulation for Exp 2-CT. Thus, it can be assumed that the residual hydroxyl groups of GR create hydrogen bonding with those of TiO_2_-SiO_2_ particles and also with the generated polar groups by plasma treatment of cotton surface, playing a positive role for anchoring particles. SEM images of Exp 4-CT and Exp 5-CT revealed some larger agglomeration of particles in the gum rosin/alginate complex matrix, while less agglomeration of particles can be seen in the case of Exp 3-CT and Exp 6-CT. The changes in morphology of Exp 1-CT…Exp 6-CT as a result of CAPP exposure are more difficult to quantify by SEM analysis due to the limited penetration of generated active species on cotton surface (in the range of few nanometers until about 200 nm) [12,34] and also by presence of surface organic additives.

**Figure 2:**
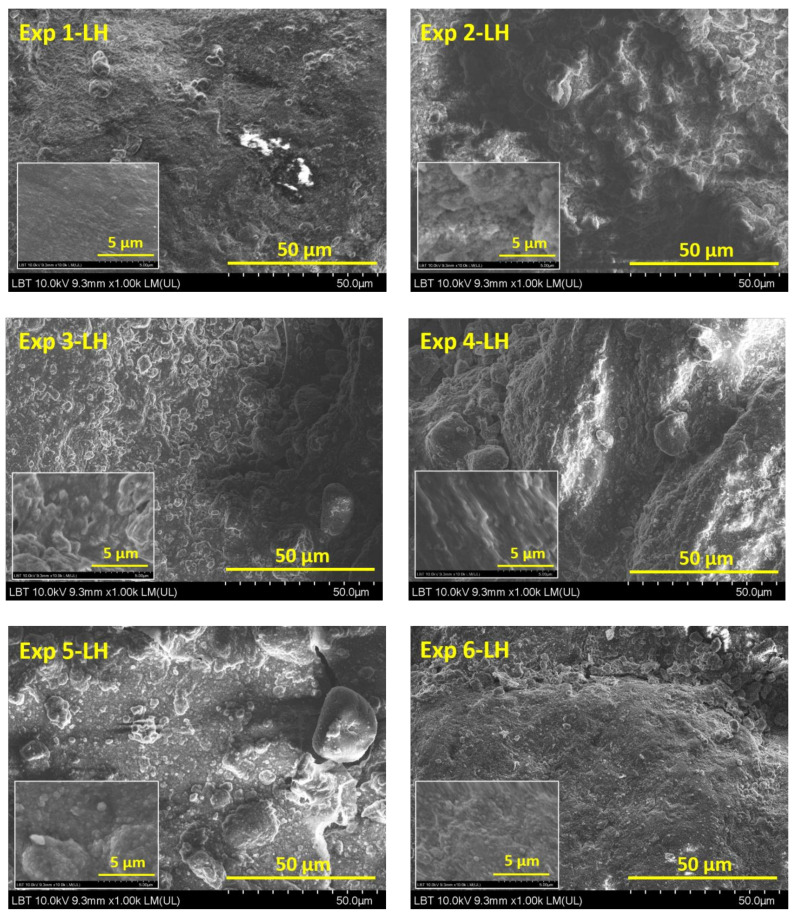
SEM images of Exp 1-LH…Exp 6-LH with magnification power of ×1000.

Leather surfaces (Exp 1-LH…Exp 6-LH) showed similar aspects (Figure 2). The organic dispersion media covered all leather surfaces. The most agglomerations of particles can be observed on Exp 1-LH, Exp 4-LH and Exp 5-LH surfaces, suggesting that these particles are not uniformly distributed at the nanometric level. No obvious plasma effect on leather surfaces was observed. However, according with earlier research described [35,36], plasma treatment causes an etching effect on the leather surface related to the modification of leather micro-pores providing better penetration or adhesion of the coating formulations; thus, the micro-pores could be partially or totally covered [30,37].

**Figure 3:**
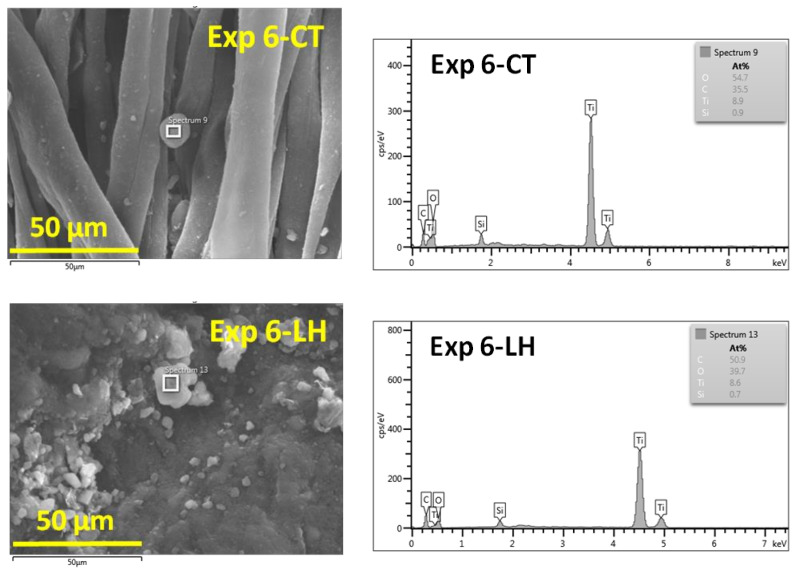
EDX patterns (**left**) of cotton and leather surfaces (**right**) modified with TiO_2_-SiO_2_-reduced graphene oxide in ethyl L-lactate matrix.

The EDX spectra of the selected Exp 6-CT and Exp 6-LH (Figure 3) showed the presence of C, O, Si and Ti. The amount of Ti and Si measured by EDX presents a relative value due to the roughness of the surfaces, which makes it difficult to calculate exactly the content of elements.

**Figure 4:**
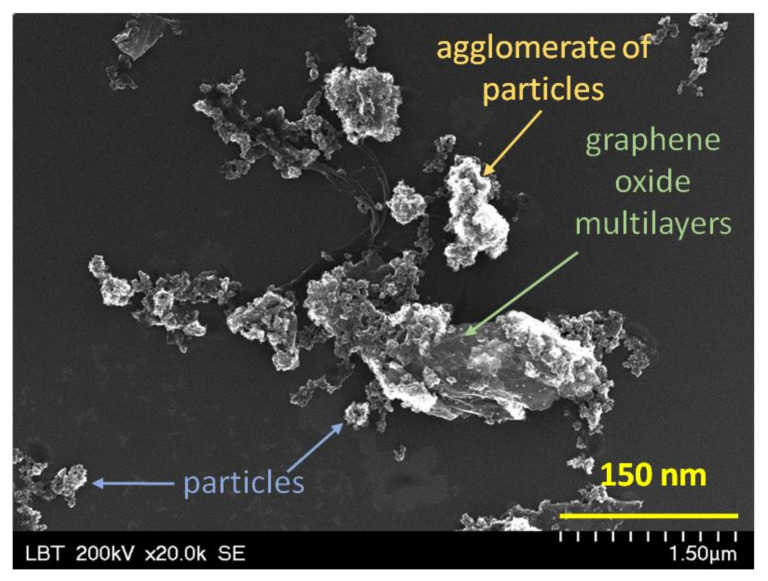
TS/GR composite morphology shown by SEM image at ×20,000 magnification.

The SEM investigation does not allow differentiation between crystalline and non-crystalline materials, TiO_2_ and SiO_2_ particles having similar appearance (size, shape, brightness and contrast). Moreover, a clear and distinct image of wrinkles of graphene oxide layers is difficult to observe. For this reason, Figure 4 presents the SEM image of TS/GR composite in order to demonstrate the presence of all constituents.

**Figure 5:**
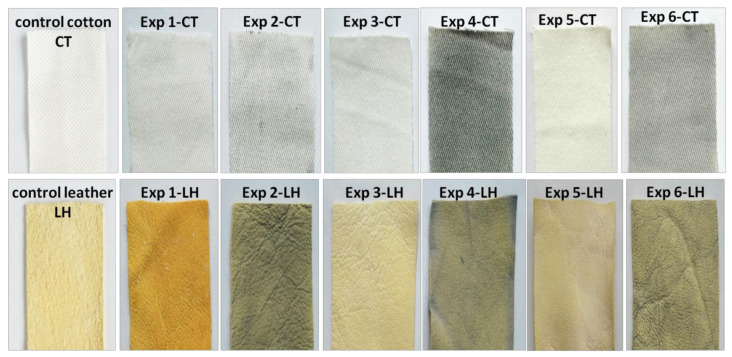
Digital photography of cotton (Exp 1-CT…Exp 6-CT) top row and leather (Exp 1-LH…Exp 6-LH) (bottom row) samples compared with control cotton (CT) and leather (LH) samples.

Taken into account the irregular cotton surface and the wrinkled leather surface, an uneven distribution of the nanopowders is likely to be achieved, as can be seen in Figure 5.

### 3.2. XRD Characterization

**Figure 6:**
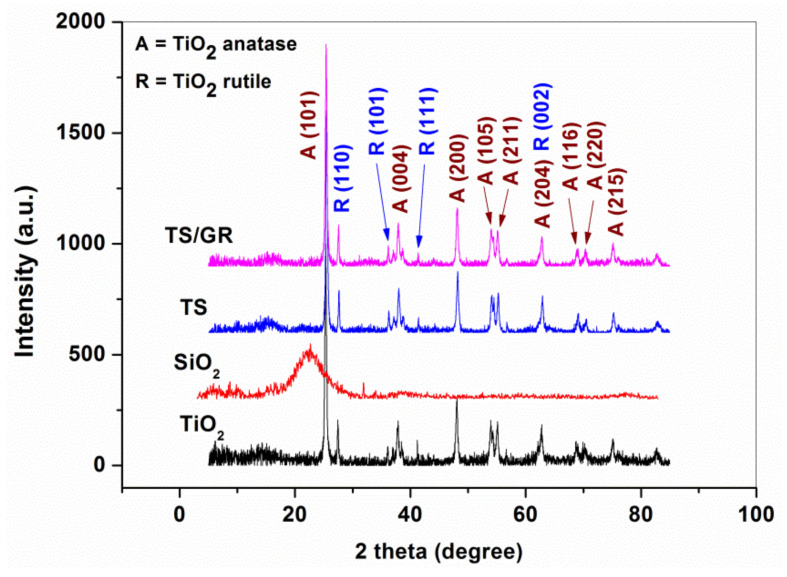
XRD spectra of pure TiO_2_, SiO_2_, TiO_2_-SiO_2_ and TiO_2_-SiO_2_/reduced graphene oxide.

The XRD spectra of pure TiO_2_, SiO_2_, TiO_2_-SiO_2_ and TiO_2_-SiO_2_/reduced graphene oxide are presented in Figure 6. The diffraction pattern of pure SiO_2_ powder revealed a low-intensity broad peak located at 2θ = 22°, indicating the amorphous state of pure SiO_2_ powder, in agreement with the previous findings [38,39]. Meanwhile, the XRD spectra of the TS and TS/GR composites showed a similar profile to the pure TiO_2_, indicating the presence of the anatase (PDF 01-078-2486) and rutile (PDF 01-078-2485) crystalline phases of TiO_2_. No contribution of SiO_2_ is observed in the diffraction pattern of TS and TS/GR composites.

**Figure 7:**
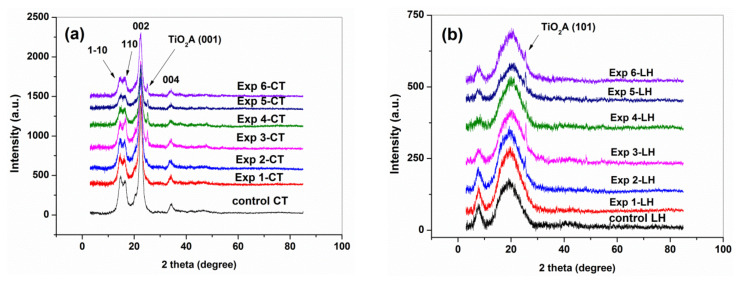
XRD pattern of (**a**) cotton and (**b**) leather samples compared to the control samples (CT and LH).

The XRD pattern of Exp 1-CT…Exp 6-CT (Figure 7a) and Exp 1-LH…Exp 6-LH (Figure 7b) indicated that the structural integrities of cellulose and collagen were retained.

The XRD pattern of all Exp-CT samples showed the characteristic diffraction peaks of typical cellulose I crystalline form that appear at 2θ = 14.6°, 16.3°, 22.6° and 33.9° corresponding to the reflections 1-10, 110, 002 and 004 [40,41].

After the normalization of XRD spectra (not shown here), the Segal’s equation [42], Equation (2), was used to determine the percentage of crystalline cellulose or the X-ray crystallinity index (CrI_XRD_) of all cotton samples:

(2) $${\mathrm{CrI}}_{\mathrm{XRD}}\;\left(\%\right)=\frac{\mathrm{Imax}\;\left(002\right)-\mathrm{Iam}}{\mathrm{Imax}\;\left(002\right)}\times 100$$

where Imax (002) is the maximum intensity at the 002 reflection of the cellulose I type peak at 2θ = 22.62°, and Iam is the intensity diffraction of the minimum or “valley” attributed to amorphous cellulose at 2θ = 18.00°.

A slight decrease of the crystallinity index was observed for all cotton samples compared with that of CT (Table 2), the relatively less crystalline structure of Exp1-CT…Exp 6-CT being associated with the increasing of water absorbed by the amorphous cellulose phase [43].

Some authors reported that the crystallinity of cotton fibers increases after plasma treatment by removing the amorphous region [44,45,46]. However, no significant change after the CAPP treatments was found, probably due to the plasma conditions as well as the presence of coating formulation on surfaces.

Compared with the control leather sample, the Exp 1-LH…Exp 6-LH samples still revealed characteristic diffraction peaks of collagen structure: a sharp peak at 7.7° corresponding to the intermolecular lateral packing of the collagen molecules and a wide one at 20.3° related to the amorphous phase of collagen caused by a loss of structural order due to unfolded collagen chains [47,48]. Based on the XRD data, the Bragg equation (Equation (3): d(Å) = λ/2sin θ, where λ is the X-ray wavelength of 1.54 Å, and θ is the Bragg scattering angle) was used to calculate the minimum values (d) of the repeated spacings from the sharp peak at 7.7° (d_7.7°_) and the diameter of the triple-helix collagen molecule from the wide peak at 20.3° (d_20.3°_). The obtained values shown in Table 3 are similar to those found in other studies, describing the dehydrated state of collagen molecules [49,50].

The X-ray diffraction data correspond to the structure of type I collagen [51] for Exp 1-LH…Exp 6-LH, indicating the preservation of leather substrate. However, a loss of structural water from the collagen and a rearrangement of inter-molecular bonding, electrostatic or van der Waals interactions could appear by the applied treatments on leather surface.

### 3.3. ATR FTIR Spectroscopy Characterization

**Figure 8:**
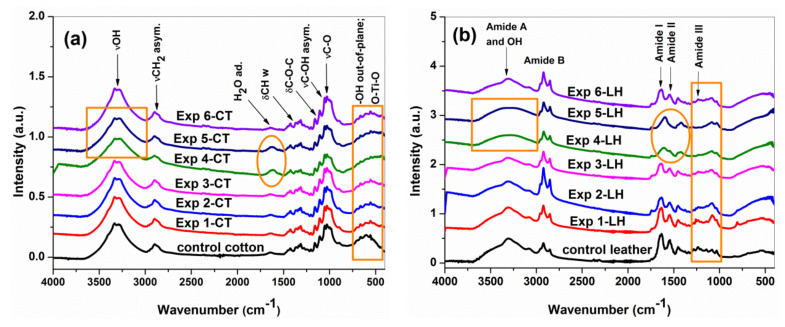
ATR-FTIR spectra of (**a**) cotton and (**b**) leather samples compared with those of control cotton and leather samples.

In consistence with XRD results, the ATR-FTIR spectra (Figure 8a,b) revealed that the cellulose and collagen structures appear as dominant components.

In agreement with previous studies [41,52,53], the Exp 1-CT…Exp 6-CT samples (Figure 8a) showed the characteristics absorption bands of cellulose: between 3000 and 3600 cm^−1^ (intra- and inter-molecular hydrogen bonding), at 2900 cm^−1^ (−CH_2_ asymmetric stretching of long alkyl chain), at 1628 cm^−1^ (adsorbed H_2_O), at 1429 cm^−1^ (−CH wagging, in-plane bending), at 1311 cm^−1^ (−CH wagging), at 1160 and 1106 cm^−1^ (asymmetric bridge C–O–C), 1053 cm^−1^ (C–OH asymmetric in-plane ring stretching of C_3_–OH), at 1029 cm^−1^ (C–O stretching of C_6_–OH), at 999 cm^−1^ (C–O stretching), at 895 cm^−1^ (C_1_–O–C_4_ asymmetric out-of-phase ring stretching; β glycosidic bond) and at 600 cm^−1^ (O–H out-of-plane bending). Although the spectra of Exp 1-CT…Exp 6-CT are quite similar to that of the control cotton sample, some changes could be observed, i.e., a slight band broadening in the range of 3000–3600 cm^−1^ (suggesting the contribution of inter-molecular hydrogen bonded −OH group from alginate structure) for all samples [54], a small increasing of band intensity at 1628 cm^−1^ (associated to the amount of bound water) for Exp 4-CT and Exp 5-CT and a widening of the band at 600 cm^−1^ (by contribution of symmetric stretching vibration of O–Ti–O groups) for all leather samples [29]. No typical absorption bands corresponding to stretching (at ~800 cm^−1^) and bending (~1000 cm^−1^) vibration modes of the Si–O–Si bonds were found due to overlapping on the fingerprint of the carbohydrate (C–O stretching) region (1160–895 cm^−1^) from cellulose. The ATR-FTIR spectra do not reveal peak shifting or new peaks, different from the control sample spectrum, indicating that no chemical interactions between cellulose and coating formulations occur during the applied treatments.

**Figure 9:**
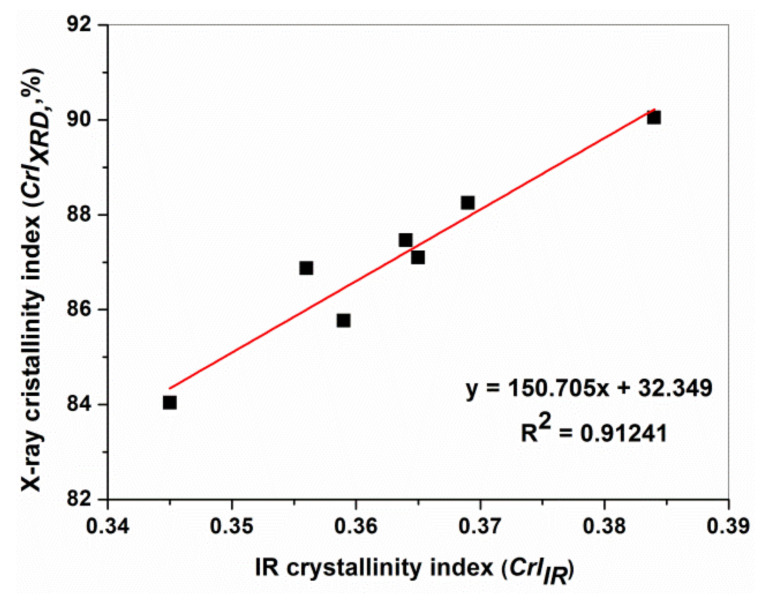
Relationship between the X-ray crystallinity index (CrI_XRD_) and the IR crystallinity ratio A_1368_/A_2898_ (*CrI_IR_*) for Exp 1…Exp 6-CT.

The ATR-FTIR findings of Exp 1-CT…Exp 6-CT corroborate relatively well with XRD results, as shown in Figure 9. The IR crystallinity index was measured using an infrared absorption ratio of A_1368(C–H bending)_/A_2898(C–H stretching)_, as proposed by Nelson and O’Connor [55], since the band at 1372 cm^−1^ is assigned to crystalline structure of cellulose, while the band at 2898 cm^−1^ is associated to the amorphous region in cellulose [34].

The ATR-FTIR spectra of all leather samples (Figure 8b) showed characteristic absorption bands assigned to the peptide bonds in collagen, namely at 3400–3300 cm^−1^ related to N–H and O–H stretching vibrations (Amide A); at 3100–2900 cm^−1^ corresponding to the asymmetric stretching of –CH_2_ groups (Amide B); 1700–1600 cm^−1^ associated with peptide’s C=O’s stretching vibration (Amide I, υ_sym_ (C=O)^major^ + ν_sym_(C–N)^minor^); 1600–1500 cm^−1^ related to C–N stretching, N–H bending vibrations (Amide II, ν_sym_(C–N) + δ(N–H)_out of phase_); 1300–1200 cm^−1^ corresponding to C–N stretching vibrations, N–H bending vibrations and wagging vibrations of CH_2_ groups from glycine backbone and proline side chains (Amide III, ν_sym_(C–N) + δ(N–H)_in phase +_ ν_w_(CH_2_)). These results are in agreement with the literature’s data [36,47,56,57], indicating that Exp 1-LH…Exp 6-LH maintained the same characteristics of native collagen.

As previously shown [58], the modification of collagen structure can be estimated based on the spectral parameter’s Amide III/1450 peak ratio and the difference between Amide I and II frequencies (∆υ = υ_1_ − υ_2_). Table 4 presents the value of these parameters calculated from the normalized ATR-FTIR spectra of the control leather and Exp 1-LH…Exp 6-LH.

The ratios of mean absorbance Amide III (at 1235 cm^−1^) and 1450 cm^−1^ were greater than 0.8, and ∆υ values were lower than 100 cm^−1^ for Exp 1-LH, Exp 2-LH, Exp 3-LH and Exp 6-LH, demonstrating the preservation of collagen triple-helix integrity [56,58,59]. The values of the Amide III/1450 peak ratio close to 0.5 suggest a modification (denaturation) of three-dimensional structure of the collagen type I [59] for Exp 4-LH and Exp 5-LH. However, the applied treatments on leather surfaces could affect the band profile of samples without damaging the collagen structure, as already proven by SEM and XRD analyses. ATR-FTIR spectra of Exp 4-LH and Exp 5-LH showed a larger band at 3300 cm^−1^ corresponding to extensive intermolecular hydrogen bonding −OH groups and a spectral absorption modification between 1770 and 1730 cm^−1^ due to the contribution of the stretching carboxylate groups from the alginate structure [54,60]. Moreover, a slight spectral difference occurs between 1300 and 1000 cm^−1^ for all final leather samples by increasing in intensity and widening of band at around 1080 cm^−1^ that may be attributed to Si–O–Si stretching vibration from the SiO_2_ structural network. The band centered at 550 cm^−1^ is due to O–Ti–O vibrations from the TiO_2_ network [61]. By comparing the spectral parameters of Exp 1-LH…Exp 4-LH (with plasma treatment) and Exp 5-LH and Exp 6-LH (without plasma treatment), it can be shown that the triple-helix conformation of collagen is not destroyed under cold plasma effect [62]. All these observations from ATR-FTIR spectra of final leather samples suggest physical interactions between collagen and coating formulations, probably by hydrogen bonding among carboxyl, amino and hydroxyl groups.

### 3.4. UV–Vis Spectroscopy Analysis

**Figure 10:**
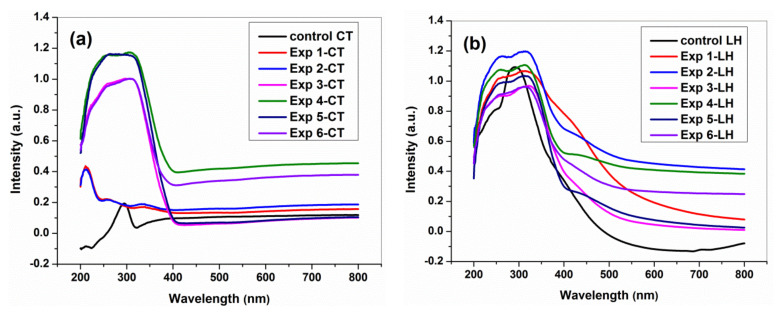
UV–Vis spectra of (**a**) Exp 1–CT…Exp 6–CT and (**b**) Exp 1–LH…Exp 6–LH in comparison with those of control cotton (CT) and leather (LH) samples.

The UV–Vis absorption spectra of Exp 1-CT…Exp 6-CT and Exp 1-LH…Exp 6-LH (Figure 10a,b) demonstrated the successful coating of cotton and leather samples. A strong increase in absorption intensity appeared in the UV region (within 200–400 nm) for Exp 1-CT…Exp 6-CT, as a result of electronic transition from the valence band (O2p) to the conduction band (Ti3d) of TiO_2_ [63], with a red shift absorption edge of TS nanopowder. Reduced graphene oxide as component of the TS/GR composite induced an increase of absorbance in the UV region from 269 nm due to the π → π* transition of aromatic C–C bond [64]. Moreover, the presence of GR on the cotton surface of Exp 2-CT, Exp 4-CT and Exp 6-CT was indicated by a broadband absorption enhancement over the whole visible spectrum (within the 400 to 800 nm range), achieved due to the surface plasmon resonance effect of the graphene unit cell, in accordance with previous results [16,63,64].

The changes in the UV–Vis spectral absorbance profile of the final cotton samples due to the CAPP effect were not observed. These findings are consistent with the observation of M.E. El-Naggar et al. [65], which reported a little shift of the maximum absorption wavelength of AgNPs-coated cotton samples by comparing the low-pressure oxygen plasma-treated (382 nm) and plasma-untreated (375 nm) cotton samples. UV–Vis spectra of Exp 4-CT and Exp 5-CT revealed a maximum absorbance at 210 nm that clearly demonstrates the formation of a calcium alginate layer on cotton surfaces.

The major component of sheepskin is type I collagen that shows an absorption peak at 240 nm attributed to the n–π* transition of carbonyl groups from peptide bonds (-CO-NH- bonds) and a more intense absorption peak at 290 nm assigned to the conjugated double bond from its side chains of the aromatic amino acids (such as phenylalanine, tyrosine and tryptophan) [66]. The presence of nanopowders on leather surfaces could be observed due to the shifting of the absorption edge toward the visible region (by enlargement of the absorption band, the so-called band “tail”) for Exp 1-LH…Exp 6-LH, more pronounced for Exp 2-LH, Exp 4-LH and Exp 6-LH containing graphene oxide. Similar to cotton samples, the UV–Vis spectra of leather samples showed no changes of their spectral profile due to the CAPP treatment.

### 3.5. Self-Cleaning Properties of the Cotton and Leather Materials

**Figure 11:**
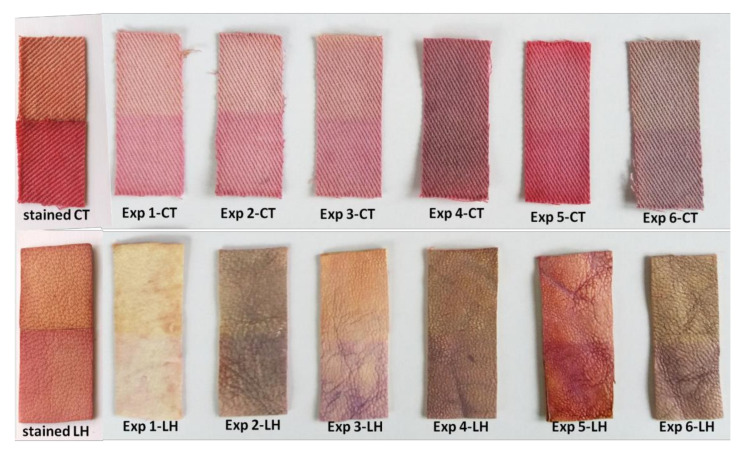
Appearances of the stained cotton (**top** row) and leather samples (**bottom** row) after 12 h of UV light irradiation (superior halves) compared with unirradiated cotton and leather samples (inferior halves).

Self-cleaning effectiveness of final cotton and leather samples was evaluated by monitoring the photo discoloration of berry juice stains from their surface, under irradiation with UV light. Figure 11 shows the color changes and lightening (fading) of stained cotton and leather samples before and after 12 h of UV light irradiation. It can be noted that the formulation coatings impart different colors to the cotton and leather samples (as seen in Figure 4). However, the loss of color during the UV exposure of cotton and leather samples was visible to the naked eye.

**Figure 12:**
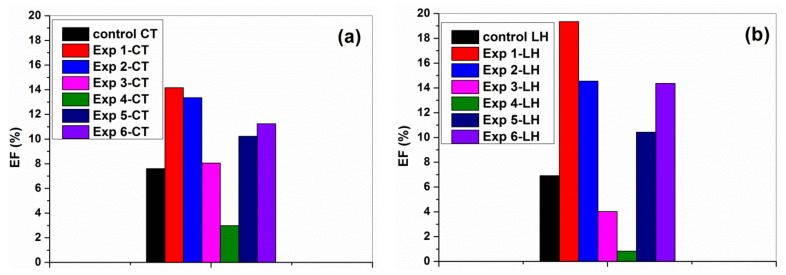
Comparative diagram of self-cleaning performance by photocatalytic activity for (**a**) cotton and (**b**) leather samples, under UV irradiation after 12 h.

Based on the spectrophotometric measurements of the stained cotton and leather samples, the decrease of absorption band intensity at 535 nm, as is typical absorption band of anthocyanins [67], was quantified, and the efficiency of degradation (%) was calculated following Equation (1). As seen in Figure 12, all samples showed photo-cleaning ability, including untreated control cotton and leather samples. This can be explained by the sensitivity towards light of anthocyanins, which cause the colors in strawberries, raspberries, blackberries and blueberries [68,69]. The observed discoloration of blueberry juice stains on the coated cotton and leather samples involved UV light sensitization of the stain dye and the photocatalytic activity of TiO_2_-SiO_2_ and TiO_2_-SiO_2_/reduced graphene oxide composites. Under UV irradiation, the photoinduced charge transfer processes at the surface of TiO_2_ lead to the generation of reactive oxygen species (ROS), such as hydroxyl radicals, HO^·^, superoxide radicals, ^1^O_2_, and hydrogen peroxide, H_2_O_2_. These ROS are then able to degrade the organic compounds during photoreduction and photo-oxidation reactions [5,70,71]. According to the literature [5,14,16,29,72,73,74], the connection between TiO_2_, SiO_2_ and reduced graphene oxide enhances the separation of generated charge carriers (electrons and holes) and facilitates the charge transfer between each other, limiting the electron–hole recombination and causing an enhanced photoactivity.

The highest photodegradation of blueberry juice stains was observed for TiO_2_-SiO_2_ nanocoating in a mixture of organosilicons and ethyl L-lactate on argon plasma pre-treated cotton (Exp 1-CT) and leather (Exp 1-LH) samples. On the opposite side, the lowest photoactivity was obtained for the formulation containing TiO_2_-SiO_2_/GR composites dispersed in GRos/alginate complex/ethyl L-lactate matrix and deposited on Ar/O_3_ plasma pre-treated cotton and also leather samples.

Similar results were reported by the D. Mihailović et al. [75] relating to the photocatalytic ability of TiO_2_ nanoparticles deposited onto polyester fabrics pre-treated with oxygen and argon plasma on the discoloration of blueberry juice stains after 24 h of UV illumination. In particular, they demonstrated that the oxygen plasma treatment of polyester surface improved the attachment of the TiO_2_ nanoparticles, ensuring superior photocatalytic effects. The same group of authors investigated the potential of corona discharge at atmospheric pressure and air RF plasma at low pressure for the cotton surface activation prior to deposition of TiO_2_ nanoparticle to improve antibacterial, UV protective and self-cleaning abilities. The results showed that both plasmas had positive effects on the overall properties of textile material, increasing the binding efficiency of TiO_2_ nanoparticles. The photocatalytic performance was confirmed by efficient discoloration of blueberry juice stain on the TiO_2_-coated cotton fabric [76]. Later study of Hui Zhang et al. [77] indicated that TiO_2_ nanoparticles can be synthesized and simultaneously loaded onto cotton fabric under the hydrothermal method using titanium sulfate and urea. Self-cleaning properties of such TiO_2_-modified cotton fibers were demonstrated on a strawberry juice stain by exposure to UV radiation for 3 h.

Summarizing, the experimental data demonstrate that the self-cleaning ability by photocatalysis of modified cotton and leather materials depends on multiple parameters, and it is clear that many pilot experiments have to be conducted to promote the development of self-cleaning applications to a large scale.

## 4. Conclusions

A new comparative study was designed to investigate the morphological, structural, optical and photo-cleaning characteristics of cotton and leather samples using TiO_2_-SiO_2_-reduced graphene oxide composites in organic dispersion media, as coating formulations and cold atmospheric pressure plasma treatments. The SEM analysis and UV–Vis spectroscopic measurements demonstrated the successful coating of cotton and leather samples. The X-ray diffraction and ATR-FTIR data revealed the typical structure of type I cellulose for cotton and type I collagen for leather substrates. Because the plasma treatment has a limited depth, its main effects are related to etching, cleaning or activation on cotton and leather surfaces, without modifying or damaging their structures. However, the optimization of plasma conditions for surface activation of different textile and leather materials with further experimentation is required. Undoubtedly, cold atmospheric pressure plasma and TiO_2_ nanoparticles modification of textile and leather surfaces demonstrated a great potential for obtaining value-added products. However, while designing the self-cleaning surface based on photocatalytic activity, multiple factors need to be taken into account, such as treatment of the surfaces, coating formulations, fabricating approaches, etc., for demanding self-cleaning applications.

## Figures and Tables

**Table 1:** Assignment of L6, 3^2^ × 2^1^ orthogonal arrays.

Experimental Runs	Factor A Gas Plasma, 5 L·min^−1^	Factor B Organic Dispersion Medium, 5 mL	Factor C Nanopowder, 0.01 g·mL^−1^
1	Ar	D4/D5/Ethyl L-lactate	TS
2	Ar	D4/D5/Ethyl L-lactate	TS/GR
3	Ar/O_3_	Ethyl L-lactate	TS
4	Ar/O_3_	GRos/Ethyl L-lactate and NaAlg/H_2_O	TS/GR
5	No plasma	GRos/Ethyl L-lactate and NaAlg/H_2_O	TS
6	No plasma	Ethyl L-lactate	TS/GR

**Obs.** For experimental samples 4 and 5, cotton and leather surfaces were sprayed with 5 mL of 2% aqueous solution of CaCl_2_ for cross-linking and transforming into insoluble material surfaces.

**Table 2:** The crystallinity index values of Exp1-CT…Exp 6-CT compared with that of control cotton (CT).

Sample	CT	Exp 1-CT	Exp 2-CT	Exp 3-CT	Exp 4-CT	Exp 5-CT	Exp 6-CT
CrI_XRD_ (%)	90.05	87.10	85.77	88.25	87.46	84.04	86.87

**Table 3:** Calculated d values from XRD data for all leather samples.

Sample	LH	Exp 1-LH	Exp 2-LH	Exp 3-LH	Exp 4-LH	Exp 5-LH	Exp 6-LH
d_7.7°_ (nm)	1.14	1.13	1.14	1.13	1.09	1.02	1.14
d_20.3°_ (Å)	4.58	4.52	4.52	4.42	4.37	4.35	4.40

**Table 4:** ATR-FTIR characteristics of all leather samples.

Sample	A_III_/A_1450_	∆υ = υ_AI_ − υ_AII_ (cm^−1^)
LH	1.01	90
Exp 1-LH	0.99	91
Exp 2-LH	0.88	90
Exp 3-LH	0.95	93
Exp 4-LH	0.67	62
Exp 5-LH	0.49	58
Exp 6-LH	0.87	90

## Data Availability

Data are available on request from corresponding authors.

## References

[1] Miśkiewicz P. (2018). Nanotechnology in textile industry. World Sci. News.

[2] Haque M. (2019). Nano Fabrics in the 21stcentury: A review. Asian J. Nanosci. Mater..

[3] Muthukrishnan L. (2021). Nanotechnology for cleaner leather production: A review. Environ. Chem. Lett..

[4] Shah M.A., Pirzada B.M., Price G., Shibiru A.L., Qurashi A. (2022). Applications of nanotechnology in smart textile industry: A critical review. J. Adv. Res..

[5] Rashid M.M., Simončič B., Tomšič B. (2020). Recent advances in TiO_2_-functionalized textile surfaces. Surf. Interfaces.

[6] Molina J. (2016). Graphene-based fabrics, and their applications: A review. RSC Adv..

[7] Neves A.I.S., Saadi Z. (2021). Challenges of Coating Textiles with Graphene. Johns. Matthey Technol. Rev..

[8] Stanca M., Gaidau C., Alexe C.-A., Stanculescu I., Vasilca S., Matei A., Simion D., Constantinescu R.-R. (2021). Multifunctional Leather Surface Design by Using Carbon Nanotube-Based Composites. Materials.

[9] Wang Y., Zheng M., Liu X., Yue O., Wang X., Jiang H. (2021). Advanced collagen nanofibers-based functional bio-composites for high-value utilization of leather: A review. J. Sci. Adv. Mater. Devices.

[10] Monteiro W.A., Shahidi S., Wiener J. (2016). Chapter 12. Radiation effects in textile materials, radiation effects in materials. Radiation Effects in Materials.

[11] Shabbir M., Ahmed S., Sheikh J. (2020). Chapter 13. Plasma and other irradiation technologies application in textile. Frontiers of Textile Materials: Polymers, Nanomaterials, Enzymes, and Advanced Modification Techniques.

[12] Jelil R.A. (2015). A review of Scrivener Publishing LLC: Beverly, MA, USAlow-temperature plasma treatment of textile materials. J. Mater. Sci..

[13] Tudoran C., Roşu M., Coroş M. (2020). A concise overview on plasma treatment for application on textile and leather materials. Plasma Process. Polym..

[14] Yuranova T., Mosteo R., Bandara J., Laub D., Kiwi J. (2006). Self-cleaning cotton textiles surfaces modified by photoactive SiO2/TiO2 coating. J. Mol. Catal. A Chem..

[15] Liu X., Huang F., Yu W. (2013). Preparation and property of TiO2/SiO2 multilayer film on the fabric by sol-gel process. Fibers Polym..

[16] Molina J., Fernandes F., Fernández J., Pastorc M., Correia A., Souto A., Carneiro J., Teixeira V., Cases F. (2015). Photocatalytic fabrics based on reduced graphene oxide and TiO2 coatings. Mater. Sci. Eng. B.

[17] Dhineshbabu N., Arunmetha S., Manivasakan P., Karunakaran G., Rajendran V. (2016). Enhanced functional properties of cotton fabrics using TiO2/SiO2 nanocomposites. J. Ind. Text..

[18] Farouk A., Sharaf S. (2016). Sol-gel hybrid nanomaterials based on TiO2/SiO2 as multifunctional finishing for cotton fabric. Egypt. J. Chem..

[19] Rilda Y., Fadhli F., Syukri S., Alif A., Aziz H., Chandren S., Nur H. (2016). Self-Cleaning TiO2-SiO2 clusters on cotton textile prepared by dip-spin coating process. J. Teknol..

[20] Landi S., Carneiro J., Ferdov S., Fonseca A., Neves I., Ferreira M., Parpot P., Soares O., Pereira M. (2017). Photocatalytic degradation of Rhodamine B dye by cotton textile coated with SiO2-TiO2 and SiO2-TiO2-HY composites. J. Photochem. Photobiol. A Chem..

[21] Li W., Gao J., Wang L. (2017). Enhancement of durable photocatalytic properties of cotton/polyester fabrics using TiO_2_/SiO_2_ via one step sonosynthesis. J. Ind. Text..

[22] Deshpande R.H., Wasif A.I., Shah K. (2019). Multifinishing of cotton using reduced graphene oxide. Int. Res. J. Eng. Technol..

[23] Shaheen T., Zaghloul S., Salem S. (2019). A new facile strategy for multifunctional textiles development through in situ deposition of SiO_2_/TiO_2_ nanosols hybrid. Ind. Eng. Chem. Res..

[24] Trang P.T.T., Giang L.H., Manh N.B., Cong T.D., Tung N.T., Anh V.T. (2021). High Flame Retardant Performance of SiO2-TiO2 Sol Coated on Polyester/Cotton Fabrics. VNU J. Sci. Nat. Sci. Technol..

[25] Gaidau C., Petica A., Ignat M., Popescu L.M., Piticescu R.M., Tudor I.A., Piticescu R.R. (2017). Preparation of silica doped titania nanoparticles with thermal stability and photocatalytic properties and their application for leather surface functionalization. Arab. J. Chem..

[26] Kaygusuz M.K., Meyer M., Aslan A. (2017). The Effect of TiO2-SiO2 Nanocomposite on the Performance Characteristics of Leather. Mater. Res..

[27] Kaygusuz M., Ide S., Karaarslan D. (2020). Nanoscopic investigation on TiO2-SiO2-GLYMO nanocomposite coated and plasma treated leathers. Polym. Technol. Mater..

[28] Kale M.B., Luo Z., Zhang X., Dhamodharan D., Divakaran N., Mubarak S., Wu L., Xu Y. (2019). Waterborne polyurethane/graphene oxide-silica nanocomposites with improved mechanical and thermal properties for leather coatings using screen printing. Polymer.

[29] Gao J., Li W., Zhao X., Wang L., Pan N. (2019). Durable visible light self-cleaning surfaces imparted by TiO_2_/SiO_2_/GO photocatalyst. Text. Res. J..

[30] Kaygusuz M., Meyer M., Junghans F., Aslan A. (2018). Modification of leather surface with atmospheric pressure plasma and nano-finishing. Polym. Plast. Technol. Eng..

[31] Bolboacă S.D., Jäntschi L. (2007). Design of Experiments: Useful Orthogonal Arrays for Number of Experiments from 4 to 16. Entropy.

[32] Pogacean F., Socaci C., Pruneanu S., Biris A., Coros M., Magerusan L., Katona G., Turcu R., Borodi G. (2015). Graphene based nanomaterials as chemical sensors for hydrogen peroxide—A comparison study of their intrinsic peroxidase catalytic behavior. Sens. Actuators B Chem..

[33] Tudoran C. (2019). Universal Cold Plasma Applicator to Be Used in Surface Engineering. Romanian Patent.

[34] Bhat N., Netravali A., Gore A., Sathianarayanan M., Arolkar G., Deshmukh R. (2011). Surface modification of cotton fabrics using plasma technology. Text. Res. J..

[35] Idris A., Majidnia Z., Valipour P. (2013). Antibacterial improvement of leather by surface modification using corona discharge and silver nanoparticles application. J. Sci. Technol..

[36] Silvestre C.R., Blasco M.P.C., López S.R., Aguilar H.P., Limiñana M.P., Gil E.B., Calpena E.O., Ais F.A. (2021). Hydrophobic Leather Coating for Footwear Applications by a Low-Pressure Plasma Polymerisation Process. Polymers.

[37] Jiang Y., Li J., Liu F., Zhang Z., Li Z., Yang M., Li L. (2018). The effects of surface modification using O_2_ low temperature plasma on chrome tanning properties of natural leather. J. Ind. Text..

[38] Martínez J., Palomares-Sánchez S., Ortega-Zarzosa G., Ruiz F., Chumakov Y. (2006). Rietveld refinement of amorphous SiO2 prepared via sol–gel method. Mater. Lett..

[39] Il’Ves V.G., Zuev M.G., Sokovnin S.Y. (2015). Properties of Silicon Dioxide Amorphous Nanopowder Produced by Pulsed Electron Beam Evaporation. J. Nanotechnol..

[40] Park S., Baker J., Himmel M., Parilla P., Johnson D. (2010). Cellulose lose crystallinity index: Measurement techniques and their impact on interpreting cellulase performance. Biotechnol. Biofuels.

[41] Abidi N., Manike M. (2017). X-ray diffraction and FTIR investigations of cellulose deposition during cotton fiber development. Text. Res. J..

[42] Segal L., Creely J.J., Martin A.E., Conrad C.M. (1959). An Empirical Method for Estimating the Degree of Crystallinity of Native Cellulose Using the X-ray Diffractometer. Text. Res. J..

[43] Cabrales L., Abidi N. (2019). Kinetics of Cellulose Deposition in Developing Cotton Fibers Studied by Thermogravimetric Analysis. Fibers.

[44] Parikh D., Thibodeaux D., Condon B. (2007). X-ray crystallinity of bleached and cross linked cottons. Text. Res. J..

[45] Karahan H., Özdo E. (2008). Improvements of surface functionality of cotton fibers by atmospheric plasma treatment. Fiber. Polym..

[46] Liu Y., Thibodeaux D., Bauer P., Van Derveer D. (2012). Comparative investigation of Fourier Transform Infrared (FT-IR) spectroscopy and X-ray Diffraction (XRD) in the determination of cotton fiber crystallinity. Appl. Spectrosc..

[47] Andonegi M., Irastorza A., Izeta A., Cabezudo S., De La Caba K., Guerrero P. (2020). A Green Approach towards Native Collagen Scaffolds: Environmental and Physicochemical Assessment. Polymers.

[48] Gao D., Cheng Y., Wang P., Li F., Wu Y., Lyu B., Ma J., Qin J. (2020). An eco-friendly approach for leather manufacture based on P(POSS-MAA)-aluminum tanning agent combination tannage. J. Clean. Prod..

[49] Sun L., Hou H., Li B., Zhang Y. (2017). Characterization of acid- and pepsin-soluble collagen extracted from the skin of Nile tilapia (*Oreochromis niloticus*). Int. J. Biol. Macromol..

[50] Xu Z., Guan X., Liu J., Fan H., Chen Y. (2017). Improving collagen extraction through an alternative strategy based on succinic anhydride pretreatment to retain collagen’s triple-helix structure. J. Appl. Polym. Sci..

[51] Orgel J.P.R.O., Antonio J.D.S., Antipova O. (2010). Molecular and structural mapping of collagen fibril interactions. Connect. Tissue Res..

[52] Ahmad I., Kan C.-W., Yao Z. (2019). Photoactive cotton fabric for UV protection and self-cleaning. RSC Adv..

[53] Stanisławska A., Staroszczyk H., Szkodo M. (2020). The effect of dehydration/rehydration of bacterial nanocellulose on its tensile strength and physicochemical properties. Carbohydr. Polym..

[54] da Silva Fernandes R., Moura M., Glenn G., Aouada F. (2018). Thermal, microstructural, and spectroscopic analysis of Ca2+ alginate/clay nanocomposite hydrogel beads. J. Mol. Liq..

[55] Nelson M., O’Connor R. (1964). Relationship of certain infrared bands to cellulose crystallinity and crystal lattice type. Part II. A new infrared ratio for estimating of crystallinity in cellulose I and II. J. Appl. Polym. Sci..

[56] Riaz T., Zeeshan R., Zarif F., Ilyas K., Muhammad N., Safi S.Z., Rahim A., Rizvi S.A.A., Rehman I.U. (2018). FTIR analysis of natural and synthetic collagen. Appl. Spectrosc. Rev..

[57] León-López A., Fuentes-Jiménez L., Hernández-Fuentes A., Campos-Montiel R., Aguirre-Álvarez G. (2019). Hydrolysed collagen from sheepskins as a source of functional peptides with antioxidant activity. Int. J. Mol. Sci..

[58] Albu M.G., Ghica M.V., Leca M., Popa L., Borlescu C., Cremenescu E., Giurginca M., Trandafir V. (2010). Doxycycline Delivery From Collagen Matrices Crosslinked With Tannic Acid. Mol. Cryst. Liq. Cryst..

[59] Júnior Z.S.S., Botta S.B., Ana P.A., Franca C.M., Fernandes K.P.S., Mesquita-Ferrari R.A., Deana A., Bussadori S.K. (2015). Effect of papain-based gel on type I collagen—spectroscopy applied for microstructural analysis. Sci. Rep..

[60] Siqueira P., Siqueira E., De Lima A.E., Siqueira G., Pinzón-Garcia A.D., Lopes A.P., Segura M.E.C., Isaac A., Pereira F.V., Botaro V.R. (2019). Three-Dimensional Stable Alginate-Nanocellulose Gels for Biomedical Applications: Towards Tunable Mechanical Properties and Cell Growing. Nanomaterials.

[61] Ojstršek A., Fakin D. (2019). Washing durability and photo-stability of nano TiO_2_-SiO_2_ coatings exhausted onto cotton and cotton/polyester fabrics. Coatings.

[62] Samouillan V., Merbahi N., Yousfi M., Gardou J.-P., Delaunay F., Dandurand J., Lacabanne C. (2012). Effect of low-temperature plasma jet on thermalstability and physical structure of type I collagen. IEEE Trans. Plasma Sci..

[63] Zhou K., Zhu Y., Yang X., Jiang X., Li C. (2011). Preparation of graphene-TiO_2_ composites with enhanced photocatalytic activity. New J. Chem..

[64] Zhang Y., Ma H.-L., Zhang Q., Peng J., Li J., Zhai M., Yu Z.-Z. (2012). Facile synthesis of well-dispersed graphene by γ-ray induced reduction of graphene oxide. J. Mater. Chem..

[65] Abdelrahman M.E.-N.T.K.M., Hatshan M. (2021). Development of antimicrobial, UV blocked and photocatalytic self-cleanable cotton fibers decorated with silver nanoparticles using silver carbamate and plasma activation. Cellulose.

[66] Ju H., Liu X., Zhang G., Liu D., Yang Y. (2020). Comparison of the Structural Characteristics of Native Collagen Fibrils Derived from Bovine Tendons Using Two Different Methods: Modified Acid-Solubilized and Pepsin-Aided Extraction. Materials.

[67] Zyoud A.H., Saleh F., Helal M.H., Shawahna R., Hilal H.S. (2018). Anthocyanin-Sensitized TiO_2_ Nanoparticles for Phenazopyridine Photodegradation under Solar Simulated Light. J. Nanomater..

[68] Verduin J., den Uijl M., Peters R., van Bommel M. (2020). Photodegradation products and their analysis in food. Food Sci. Nutr..

[69] Laleh G., Frydoonfar H., Heidary R., Jameei R., Zare S. (2006). The Effect of Light, Temperature, pH and Species on Stability of Anthocyanin Pigments in Four Berberis Species. Pak. J. Nutr..

[70] Bozzi A., Yuranova T., Guasaquillo I., Laub D., Kiwi J. (2005). Self-cleaning of modified cotton textiles by TiO_2_ at low temperatures under daylight irradiation. J. Photochem. Photobiol. A Chem..

[71] Radetić M. (2013). Functionalization of textile materials with TiO_2_ nanoparticles. J. Photochem. Photobiol. C Photochem. Rev..

[72] Zhang Q., Bao N., Wang X., Hu X., Miao X., Chaker M., Ma D. (2016). Advanced Fabrication of Chemically Bonded Graphene/TiO_2_ Continuous Fibers with Enhanced Broadband Photocatalytic Properties and Involved Mechanisms Exploration. Sci. Rep..

[73] Padmanabhan N.T., Thomas N., Louis J., Mathew D.T., Ganguly P., John H., Pillai S.C. (2021). Graphene coupled TiO2 photocatalysts for environmental applications: A review. Chemosphere.

[74] Babyszko A., Wanag A., Sadłowski M., Kusiak-Nejman E., Morawski A.W. (2022). Synthesis and Characterization of SiO2/TiO2 as Photocatalyst on Methylene Blue Degradation. Catalysts.

[75] Mihailović D., Šaponjić Z., Molina R., Puač N., Jovančić P., Nedeljković J., Radetić M. (2010). Improved Properties of Oxygen and Argon RF Plasma-Activated Polyester Fabrics Loaded with TiO_2_ Nanoparticles. ACS Appl. Mater. Interfaces.

[76] Mihailović D., Šaponjić Z., Radoičić M., Lazović S., Jovančić C.J.B.P., Nedeljković J., Radetić M. (2011). Functionalization of cotton fabrics with corona/air RF plasma and colloidal TiO_2_ nanoparticles. Cellulose.

[77] Zhang H., Zhu L., Sun R. (2014). Structure and Properties of Cotton Fibers Modified with Titanium Sulfate and Urea under Hydrothermal Conditions. J. Eng. Fibers Fabr..

